# Molecular Characterization of *Shigella sonnei*: An Increasingly Prevalent Etiologic Agent of Shigellosis in Guizhou Province, Southwest of China

**DOI:** 10.1371/journal.pone.0156020

**Published:** 2016-05-19

**Authors:** Shijun Li, Jianping Wang, Xiaoyu Wei, Ying Liu, Lu You, Xia Luo, Guangpeng Tang, Qiangzheng Sun, Changyun Ye, Jianguo Xu, Dingming Wang

**Affiliations:** 1 Institute of Communicable Disease Control and Prevention, Guizhou Provincial Center for Disease Control and Prevention, Guiyang, Guizhou, China; 2 State Key Laboratory for Infectious Disease Prevention and Control, Beijing, China; 3 National Institute for Communicable Disease Control and Prevention, Chinese Center for Disease Control and Prevention, Beijing, China; 4 Department of Molecular and Cellular Biology, University of Guelph. Guelph, Canada; University of Minnesota, UNITED STATES

## Abstract

**Background:**

Shigellosis is a serious problem in Guizhou and *Shigella sonnei* is an increasingly prevalent etiologic agent of local shigellosis cases. No data, however, are available about the molecular characterization of the local isolates of *S*. *sonnei*. We have conducted this study to molecularly characterize the clinical isolates of *S*. *sonnei* in Guizhou Province.

**Results:**

76 *S*. *sonnei* isolates, including four isolates from 1974–1982 and 72 isolates from 2008–2010, were used for analysis in this study. Pulsed-field gel electrophoresis (PFGE) based on XbaI digestion divided the 76 isolates into 38 PFGE patterns (PT) and 15 PTs were represented by more than one isolates with PT31 (N = 8) containing the most number of isolates, followed with PT2 (N = 6). Multiple-Locus Variable number tandem repeat (VNTR) Analysis (MLVA) based on seven VNTR loci discriminated them into 19 different MLVA types (MTs), and four MTs were represented by more than one isolate with MT4 (N = 39) containing the most number of isolates, followed with MT12 (N = 18). 15 Multilocus sequence typing (MLST) base on 15 loci differentiated the isolates into six sequence types (STs), among which four STs were novel. The most common STs are ST76 (N = 43) and ST116 (N = 25), accounting for 92.1%. Correlation between genetic relationships and geographical origins or isolation years was observed among the isolates studied. Majority of isolates were clustered in accordance with the origin of isolation years based on the genetic data, which were also from similar geographical origins.

**Conclusions:**

Our results revealed the molecular characteristics including the specific genotypes such as four novel STs, clonal relationship, and genetic changes of local isolates from different years, which enhances our understanding of molecular characteristics of *S*. *sonnei* and contributes to the prevention and control of shigellosis in Guizhou Province.

## Background

The shigellae are the causative agents of shigellosis, a common diarrheal disease in developing countries [1, 2]. The main symptom of this infection is bloody diarrhoea and the minimum infective dose is as low as 10–100 bacterial cells due to relative resistance to stomach acid [3]. The infection is most frequent in children, the elderly and the immunocompromised [4]. There are about 164.7 million cases of shigellosis annually worldwide, resulting in 1,100,000 deaths, most of which are children under five years of age [2, 5].

*Shigella* can be differentiated into four species or serogroups, *S*. *dysenteriae*, *S*. *flexneri*, *S*. *boydii*, and *S*. *sonnei* based on biochemical properties and group-specific O antigens in the outer membrane of the cell wall. There is a shift in *Shigella* dominance from *S*. *flexneri* to *S*. *sonnei* in developing and developed countries [3, 6–8]. In China, an previous study on serotype distribution of *Shigella* reported that *S*. *flexneri*, from 1989 to 2010, was always the most frequently isolated serogroup, followed by *S*. *sonnei*, but after 2000, there was a significant upward trend in the constituent ratio of group *S*. *sonnei* in south China [9]. Guizhou Province, with nearly 50 million people, is an impoverished province in the southwest of China. It has been reported that *Shigella* was isolated as early as 1950s in Guizhou Province [10], and 2,266 *S*. *flexneri* isolates were isolated in Guizhou Province from 1955 to 1987 [10]. However, *S*. *sonnei* is becoming the dominant serotype causing shigellosis in Guizhou in recent years. For instance, there were 48,222 cases of human shigellosis were reported from 2007 to 2010, and 146 isolates of *Shigella*, including 102 *S*. *sonnei* and 44 *S*. *flexneri* isolates, were isolated in Guizhou [11].

Molecular typing methods are powerful tools for epidemiological investigation of *Shigella* infections as well as other bacterial infections such as those caused by Shigatoxin-producing Escherichia coli O157: H7 [2]. PFGE is the gold standard method among the molecular typing methods with a standardized PulseNet protocol, which makes it possible to compare bacterial DNA fingerprints among laboratories, even internationally [2, 12, 13]. Nevertheless, more powerful and easier methods such as multilocus variable number tandem-repeat analysis (MLVA) and multilocous sequence typing (MLST) are newly devised. MLVA is based on the inherent variability of short sequences that are organized as tandem repeats at multiple VNTR loci, which has been used to study the genetic relatedness among *S*. *sonnei* [2, 3, 14, 15]. MLST is a housekeeping gene sequence-based genotyping technique first introduced by Maiden et al., which provides reproducibility, comparability and transferability between laboratories [16, 17]. The most important advantage of MLST is the unambiguity and electronic portability of nucleotide sequence data [18]. Besides, MLST sequence data can be held through a central database and queried through a web server [19, 20]. By MLST analysis, *S*. *flexneri* were categorized into two sequence type (ST) complexes based on seven house- keeping genes [21, 22], and then MLST based on 15 house-keeping genes were applied in *Shigella* genotyping and the protocol is shared at the EcMLST website [23, 24].

Although *S*. *sonnei* is becoming an important etiologic agent of shigellosis in Guizhou Province, there is limited information on the genetic background of local strains. Therefore, the objective of the study was to molecularly characterize the local *S*. *sonnei* strains with PFGE, MLVA and MLST. In addition, the prospect of using PFGE, MLVA and MLST for routine subtyping of local *S*. *sonnei* was compared and evaluated. This was intended to aid clinical laboratory diagnosis, active surveillance, outbreak investigation and source tracking for shigellosis the locality.

## Materials and Methods

### Bacteria isolates and serological confirmation

All the *S*. *Sonnei* isolates were serologically confirmed by slide agglutination with commercial *Shigella* monovalent antisera (Denka Seiken, Japan) according to the manufacturer’s instructions [25]. The confirmed isolates were kept in brain heart infusion broth with 50% glycerol at -80°C for further study.

### PFGE

*S*. *sonnei* isolates were analyzed by PFGE with the digestion of XbaI according to the U.S. CDC PulseNet protocol [13, 26]. Briefly, XbaI-digested *Salmonella* serotype Braenderup H9812 was used as the molecular weight standard. Electrophoresis was performed with a CHEF-DR III System (Bio-Rad Laboratories, Hercules, CA) using 1% SeaKem Gold agarose. The interpretation of the PFGE patterns was performed with BioNumerics software (Applied Maths, St-Martens-Latern, Belgium) using the Dice similarity coefficient. The tree indicating relative genetic similarity was constructed on the basis of the unweighted pair group method of averages (UPGMA) and a position tolerance of 1%. A PFGE pattern with one or more DNA bands different from the others was taken to be a unique PFGE pattern. A dendrogram constructed using the PFGE patterns was generated by the UPGMA algorithm using the Dice-predicted similarity value of two patterns.

### Preparation of DNA templates

DNA templates for PCR were prepared directly from bacterial colonies by the boiling method [25]. Briefly, a single colony from an overnight culture at 37°C on LB agar was suspended in 30 μl of distilled water and boiled at 100°C for 10 min. The sample was immediately cooled on ice for 5 min and centrifuged at 13,000 × *g* at 4°C for 10 min. The supernatant, containing DNA, was used as the template for PCR amplification.

### MLVA

Seven VNTR loci, SS1, SS3, SS6, SS9, SS10, SS11, and SS13 and the primer sequences and conditions reported in previous studies were used in this study [3, 14]. Briefly, the forward primer for each primer set was labeled at its 5' end with an ABI compatible dye, HEX, FAM, TAMRA, or ROX by Tsingke (Beijing, China). Two multiplex polymerase chain reactions (mPCR) were carried out. The PCR products were analyzed by capillary electrophoresis on an ABI 3730XL sequencer with GeneScan 500 LIZ Size Standard (Applied Biosystems). Data were collected, and the lengths of the amplicons were determined with GeneScan data analysis software, v. 3.7 (Applied Biosystems). The copy number of the repeats of each VNTR locus was deduced from the length of the amplicons. This number was then converted into an allele designation, which in turn formed the allele string for the seven loci. The allele string was constructed in the following order: SS1-SS3-SS6-SS9-SS10-SS11-SS13. The data were incorporated into BioNumerics software and analyzed as described previously [27]. Each unique allelic string was designated a unique MLVA type (MT). A dendrogram was constructed by UPGMA clustering based on categorical coefficient. Minimum spanning tree algorithm was used to construct a minimum spanning tree (MST) to determine phylogenetic pattern.

### MLST

15 loci including arcA, aroE, aspC, clpX, cyaA, dNaG, fadD, grpE, icdA, lysP, mdh, mtlD, mutS, rpoS and uidA were selected based on primers as previous study described [23, 24], which can be accessed on the sharing EcMLST website (http://www.shigatox.net/ecmlst/cgi-bin/index). Following the standard MLST protocol, the PCR products were detected by electrophoresis of 3μl of each reaction on a 1.5% agarose gel for 30 min at 100 V, and were sequenced by ABI 3730XL DNA sequencer. Each allele was assigned a different allele number and the allelic profile (string of fifteen integers) was used to define the sequence type (ST). The EcMLST website was established to provide a resource to investigators who can use this to assign the ST of further strains. New allele numbers and sequence type were submitted to the EcMLST curator for allocation. The data were incorporated into BioNumerics software and a dendrogram was constructed by UPGMA clustering based on categorical coefficient, and a minimum spanning tree (MST) analysis was used to infer relationships among the isolates and was done using BioNumerics (Applied Maths, Belgium) [23].

## Results

### Distribution of isolates and serotypes

A total of 76 strains of *S*. *sonnei* isolated in Guizhou Province were used for analysis in this study. Among the 76 isolates used in this study, four isolates were collected in 1974–1982, while the remaining 72 isolates were from 2008–2010. All the isolates originated from five prefectures (including Guiyang, Anshun, Qianxinan, Qiandongnan, Zunyi) of the nine prefectures in Guizhou Province, with the exception of prefecture Bijie, Qiannan Tongren, and Liupanshui. The results of slide agglutination test showed that only 2.63% (2/76) of the isolates belonged to *S*. *sonnei* phase I isolates, while 97.37% (74/76) of the isolates were serologically identified as *S*. *sonnei* phase II isolates.

### PFGE based Genotypes

**Fig 1:**
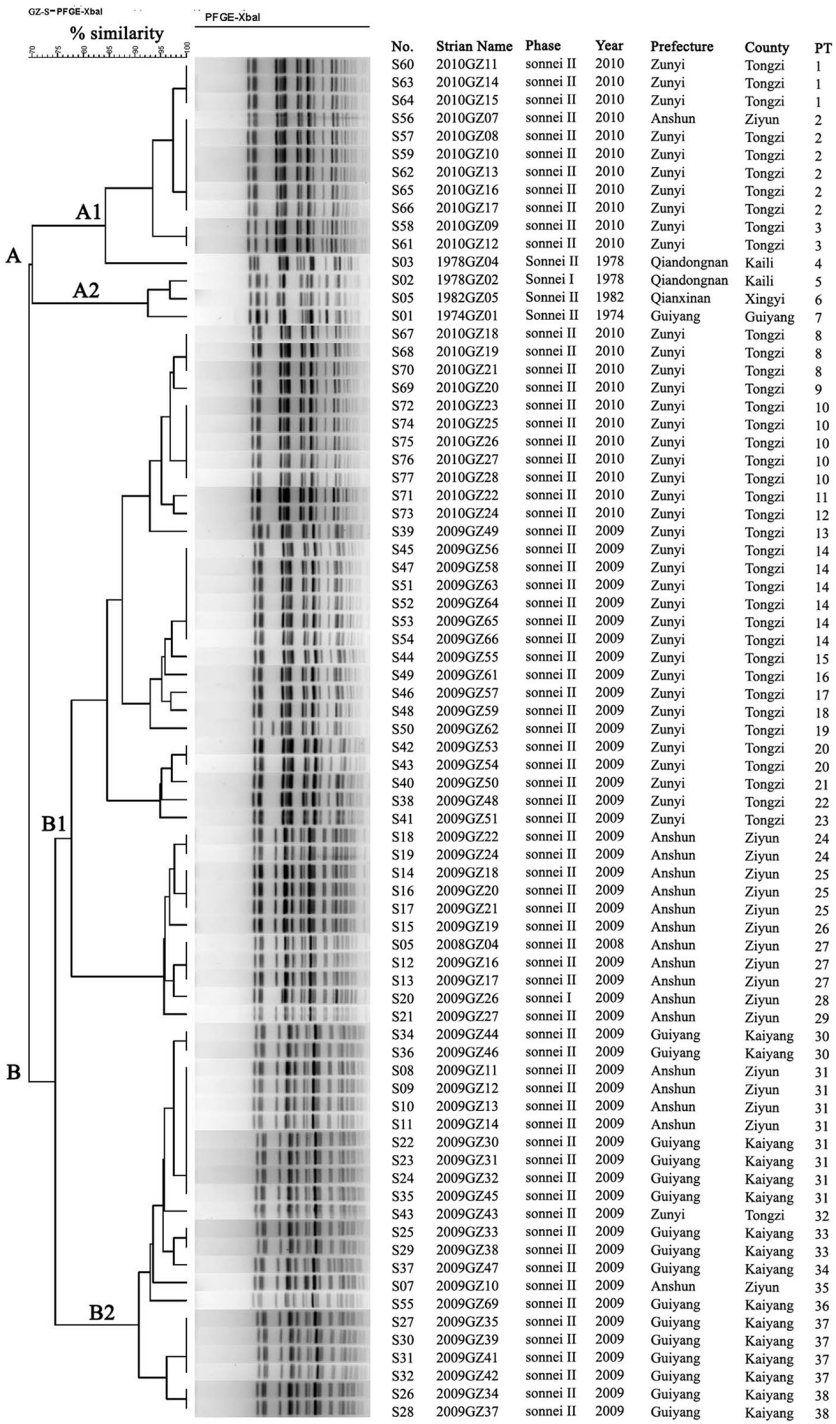
Relationships of 76 *Shigella sonnei* isolates from Guizhou Province based on Pulsed Field Gel Electrophoresis (PFGE). The 76 *S*. *sonnei* isolates from Guizhou province were analyzed by PFGE using XbaI. The interpretation of the PFGE patterns was performed with BioNumerics software using the Dice similarity coefficient. The tree indicating relative genetic similarity was constructed on the basis of the unweighted pair group method of averages (UPGMA) and a position tolerance of 1%. A PFGE pattern with one or more DNA bands different from the others was taken to be a unique PFGE pattern. The dendrogram constructed using the PFGE patterns was generated by the UPGMA algorithm using the Dice-predicted similarity value of two patterns. The corresponding PFGE pattern, serotype and background information were shown alongside the dendrogram on the right.

The genotypes and genetic relatedness of the 76 isolates were determined by PFGE. XbaI-digested *S*. *sonnei* DNA generated 38 reproducible unique PFGE patterns (PT). 15 patterns were represented by more than one isolates with PT31 (N = 8) containing the most number of isolates, followed with PT2 (N = 6). The dentrogram (Fig 1) of all the 76 isolates showed a 65% coefficient of similarity and can be classed into two gross clusters on the basis of their serotypes (cluster A and B). All the four isolates from 1974–1982 and part of the isolates from 2010 formed the cluster A, while all the isolates from 2009, part of isolates from 2012 and the one strain from 2008 were distributed in cluster B. Cluster A is further divided into cluster A1 and A2. Isolates from 2010 (cluster A1) and 1974–1982 (cluster A2) were further closely clustered in accordance with the isolation time, respectively. Cluster B was also grouped into B1 and B2 cluster. Isolates in cluster B1 included part isolates from 2009 and 2010, which were further clustered based on the origin of isolation year, whereas the one isolate (2008GZ04) from 2008 and part of isolates from 2009 were included in cluster B2. Besides, most of the isolates from the same year were closely clustered in accordance with the region origin in the dentrogram.

### MLVA based Genotypes

**Fig 2:**
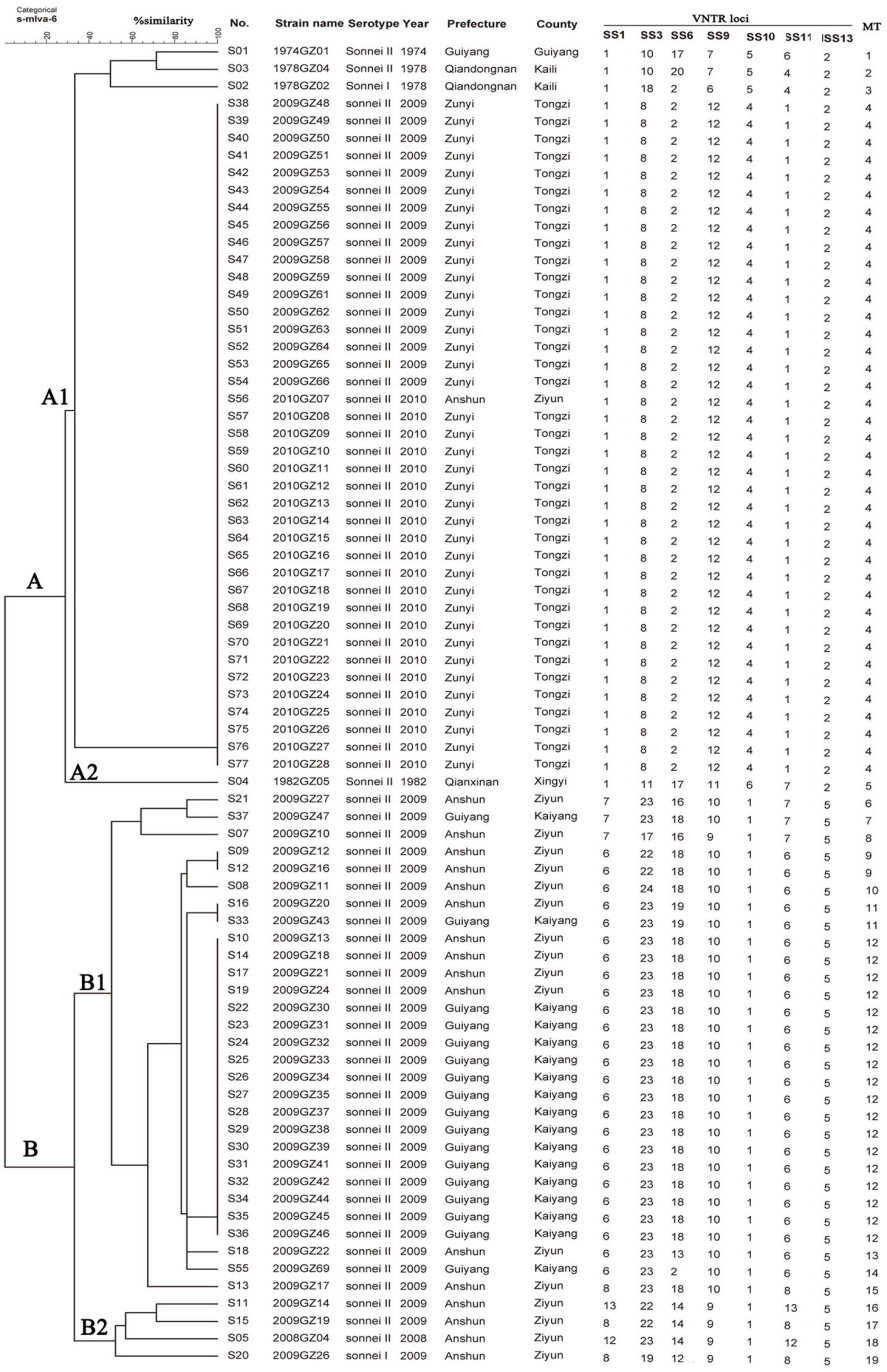
Relationships of 76 *Shigella sonnei* isolates from Guizhou Province based on the Multiple-Locus Variable number tandem repeat (VNTR) Analysis (MLVA). The 76 *S*. *sonnei* isolates from Guizhou Province were analyzed by MLVA based on seven VNTR loci. The copy number of the repeats of each VNTR locus was deduced from the length of the amplicons. This number was then converted into an allele designation, which in turn formed the allele string for the seven loci. The allele string was constructed in the following order: SS1-SS3-SS6-SS9-SS10-SS11-SS13. The data were incorporated into BioNumerics software and analyzed. Each unique allelic string was designated a unique MLVA type (MT). A dendrogram constructed using the PFGE patterns was generated by the UPGMA algorithm using the Dice-predicted similarity value of two patterns. The corresponding MLVA type with copy numbers for the seven VNTRs, serotype, and background information were shown alongside the dendrogram on the right.

**Fig 3:**
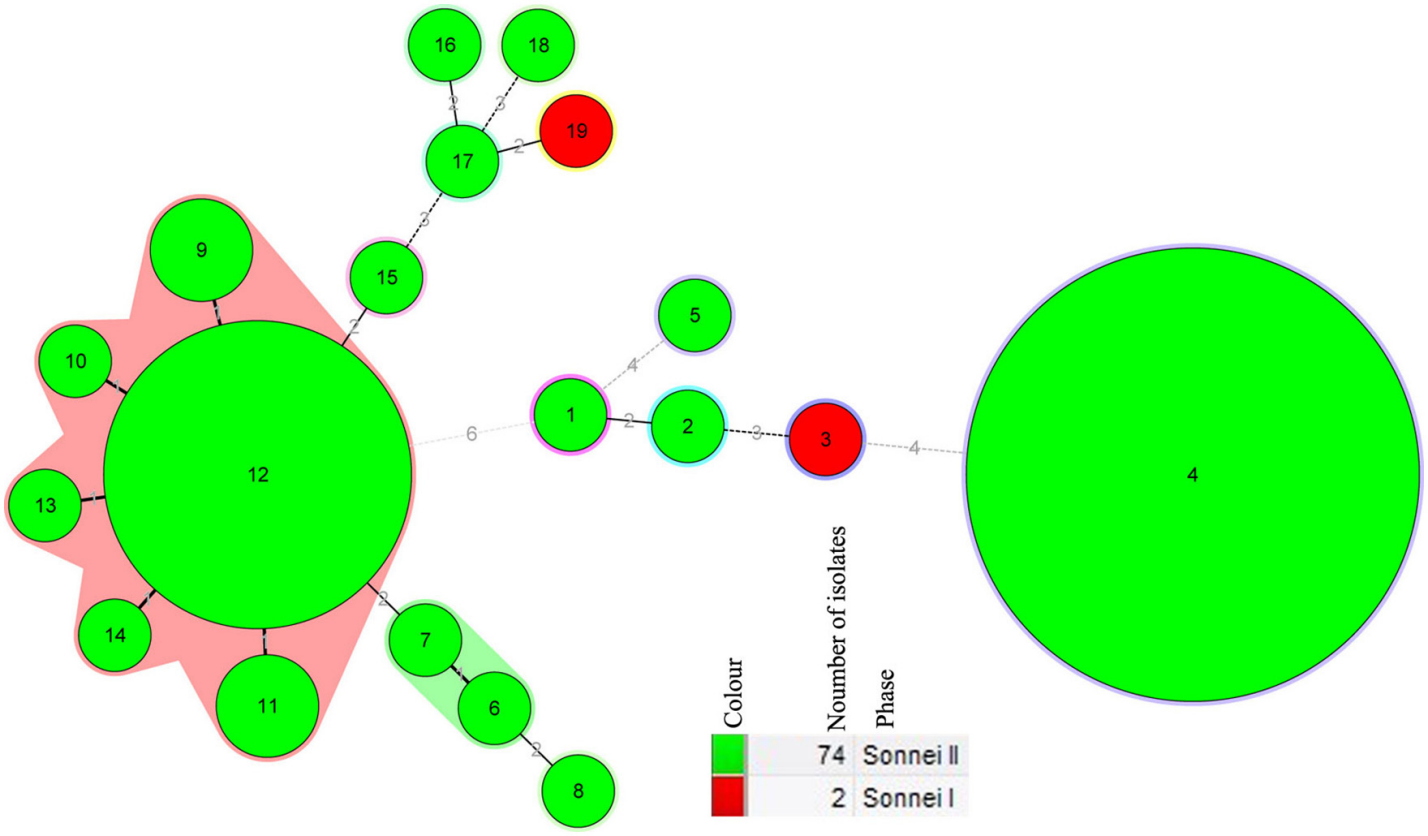
Minimum spanning tree of the isolates of 76 *S*. *sonnei* isolates from Guizhou Province based on the Multiple-Locus Variable number tandem repeat (VNTR) Analysis (MLVA). The minimum spanning tree was constructed with the MLVA profiles of 76 isolates from Guizhou Province using BioNumerics software. Minimum spanning tree algorithm of BioNumerics software was used to construct a minimum spanning tree (MST) to determine phylogenetic pattern. Each circle corresponds to a MLVA type. The shadow zones in different color correspond to different MLVA clusters (MC), including MC12 and MC7. The size of the circle is proportional to the number of the isolates, and the color within the cycles represents the serotypes of the isolates. The corresponding color, number of isolates and serotypes were shown alongside the minimum spanning tree on the right.

MLVA based on seven VNTR loci were performed to further characterize the *S*. *sonnei* isolates. All the 76 isolates showed a low coefficient of similarity (about 25.00%). The copy numbers of each VNTR locus are listed in Fig 2. The 76 *S*. *sonnei* isolates, based on the unique MLVA profiles, were discriminated into 19 different MLVA types (MTs). Four MTs were represented by more than one isolates with MT4 (N = 39) containing the most number of isolates, followed with MT12 (N = 18). M4 included 36 isolates come from 2009 and 2010, accounting for 47.37% (36/76) of the total number of isolates, while MT12 contains 18 isolates, making up 23.68% (18/76) of the total. Two main clusters, cluster A and B, were observed from the dendrogram generated (Fig 2). Cluster A was further divided into cluster A1 and A2. Three isolates (1974GZ01, 1978GZ02 and 1978GZ04) and part of isolates from 2009 and 2010 constitute the cluster A1 cluster, in which the isolates were further clustered based on the isolation years, while isolate 1982GZ05 separately formed the clade A2. All the isolates in cluster B were come from 2009, with exception of isolate 2008GZ04 from 2008. Isolates in cluster B were further gouped into cluster B1 and B2, which was further grouped in to subclusters in accordance with the region origin. The MST based on the MLVA data of 76 isolates indicated that the 19 MLVA profiles were divided in to two MLVA clusters (MC12 and MC7) and 11 singletons (Fig 3). MC12 contained isolates of six MTs (including MT9-14), while MC6 contains isolates belonged to MT6 and MT7.

### MLST based genotypes

**Fig 4:**
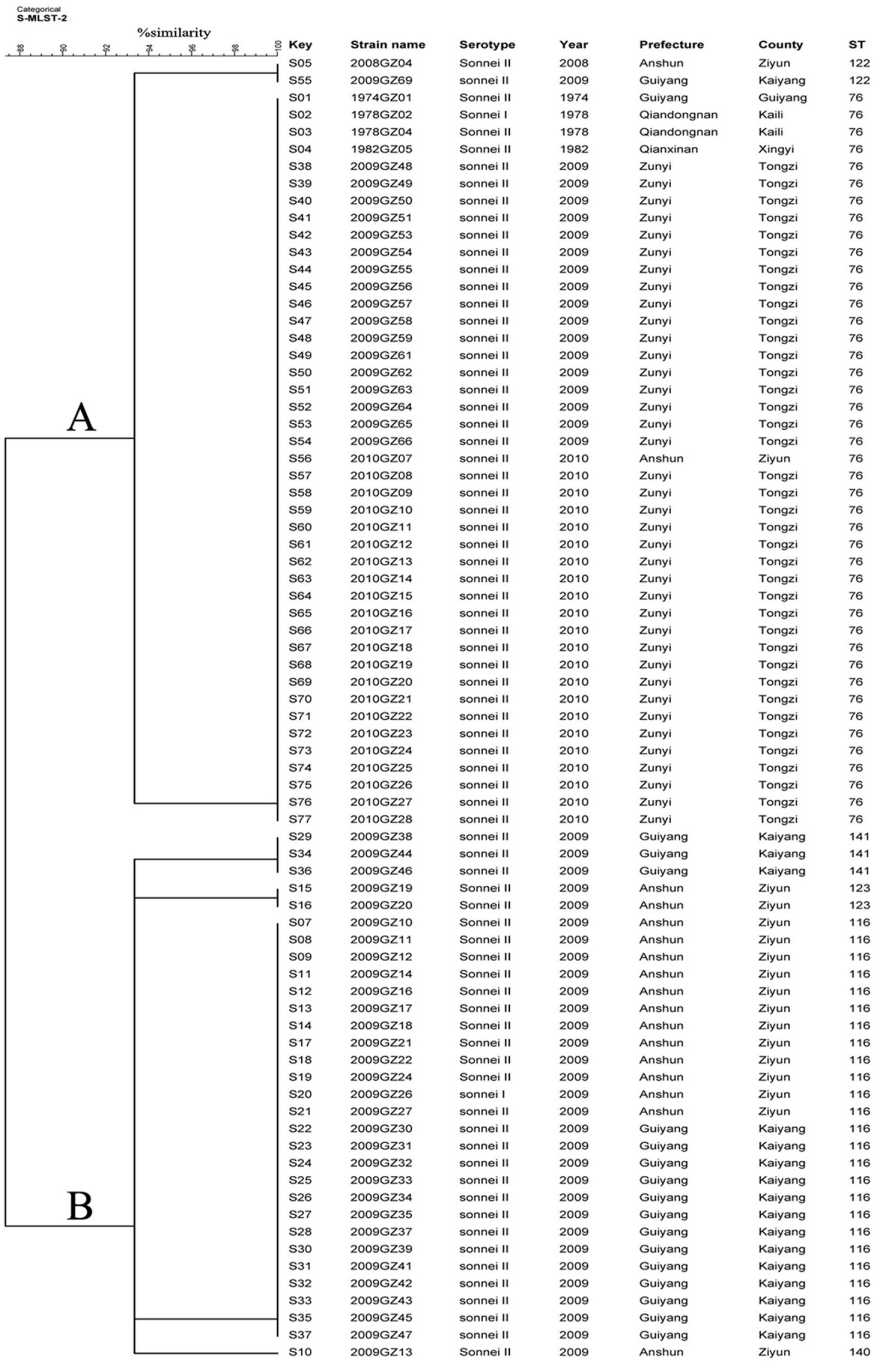
Relationships of 76 *Shigella sonnei* isolates from Guizhou Province based on Multilocus sequence typing (MLST). The 76 *S*. *sonnei* isolates from Guizhou province were analyzed by MLST based on 15 loci including arcA, aroE, aspC, clpX, cyaA, dNaG, fadD, grpE, icdA, lysP, mdh, mtlD, mutS, rpoS and uidA. The primers sequence for the 15 alles can be accessed on the sharing EcMLST website (http://www.shigatox.net/ecmlst/cgi-bin/index). The clustering tree was constructed based on the allele number of 15 loci using BioNumerics Software. The corresponding ST, serotype, and background information were shown alongside the dendrogram on the right.

**Fig 5:**
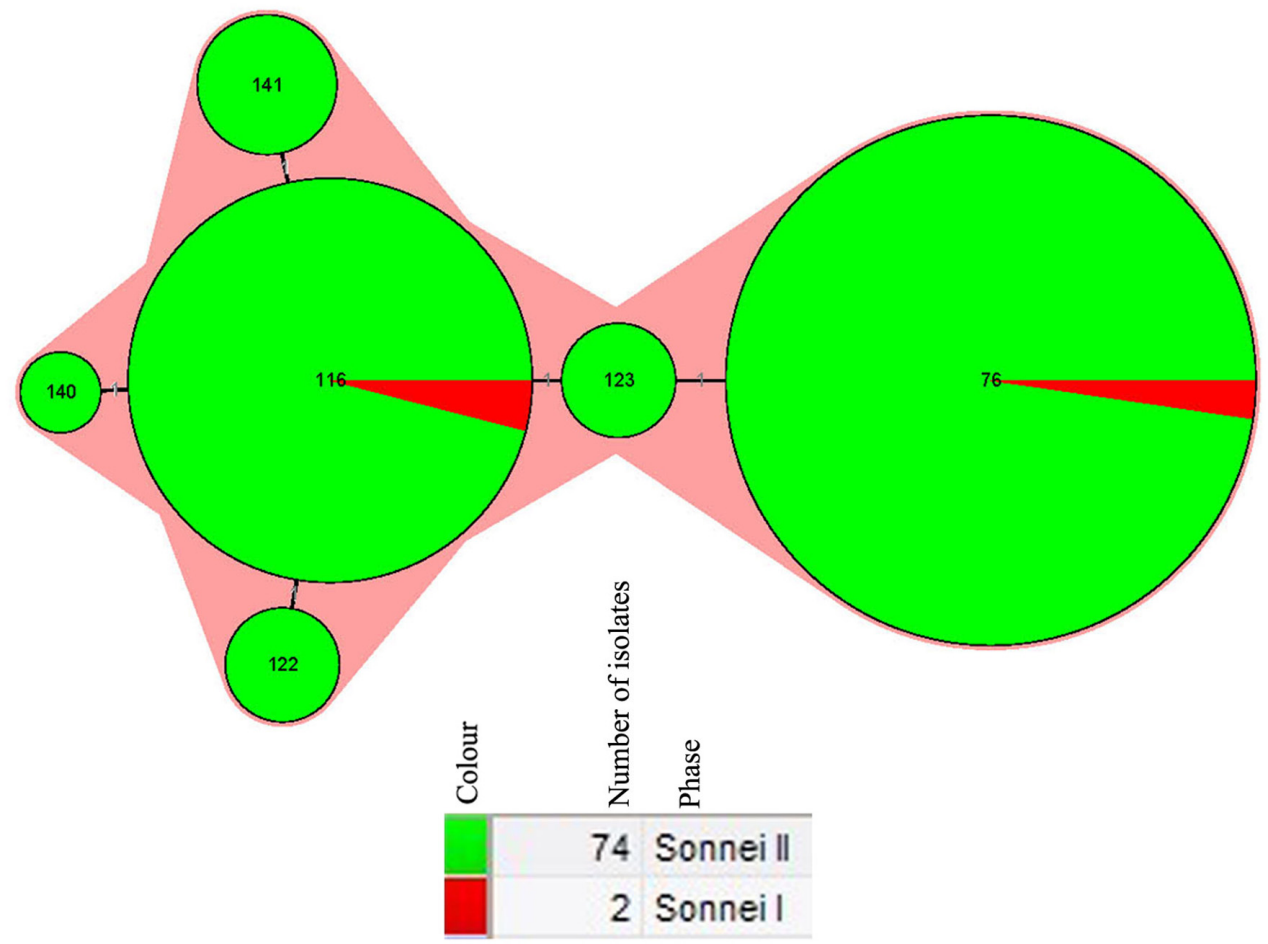
Minimum spanning tree of the isolates of 76 *S*. *sonnei* isolates from Guizhou Province based on the Multilocus sequence typing (MLST). The minimum spanning tree was constructed with the six STs of the 76 isolates from Guizhou Province using BioNumerics Software. Minimum spanning tree algorithm of BioNumerics software was used to construct a minimum spanning tree (MST) to determine phylogenetic pattern. Each circle corresponds to a sequence type. The shadow zone indicates the six STs belonging to one clonal complex. The size of the circle is proportional to the number of the isolates, and the color within the cycles represents the serotypes of the isolates. The corresponding colour, serotype, number of isolates and back ground information were shown below the minimum spanning tree.

The 76 isolates were divided into six STs, including ST76, ST116, ST122, ST123, ST140 and ST141. Among the six STs, ST76 and ST116 were previously reported, while four STs including ST122, ST123, ST140 and ST141 were novel. The allele number for each loci and the designation of ST are listed in Table 1 and shared in the EcMLST website. The most common STs are ST76 (N = 43) and ST116 (N = 25), accounting for 92.1%. ST76 contained the most number of isolates, represented by 42 isolates of phase II from different years and one isolate (1978GZ02) of phase I from 1978, accounting for 55.26% of the total isolates. ST116 includes 23 isolates of phase II and one phase I isolate (2009GZ26). All the isolates of ST116 were come from 2009, making up for 31.58% (24/76) of the total isolates. Clustering tree (Fig 4) based on the MLST data shows that all the 76 isolates were divided into two gross clusters (Cluster A and B). All the four isolates from 1974–1982, parts of isolates from 2009 and the one isolated from 2008 were grouped in cluster A, while all the isolates in cluster B were come from 2009. Both of cluster A and B were further divided into subclusters. Besides, according to the MST (Fig 5) based on the eBURST algorithm with member STs differing by only one of the 15 loci, all the six STs belonged to one clonal complexes (CCs).

**Table 1:** Results obtained using MLST for 76 *S*. *sonnei* isolates from Guizhou Province.

Strain No.	Isolates Name	Alle profiles															ST
		arcA	aroE	aspC	clpX	cyaA	dNaG	fadD	grpE	icdA	lysP	mdh	mtlD	mutS	rpoS	uidA	
S01	1974GZ01	9	13	4	19	13	3	18	3	21	14	23	13	17	17	1	76
S02	1978GZ02	9	13	4	19	13	3	18	3	21	14	23	13	17	17	1	76
S03	1978GZ04	9	13	4	19	13	3	18	3	21	14	23	13	17	17	1	76
S04	1982GZ05	9	13	4	19	13	3	18	3	21	14	23	13	17	17	1	76
S05	2008GZ04	9	13	18	19	13	3	18	3	21	14	23	13	17	17	1	122
S07	2009GZ10	9	13	18	19	13	3	18	3	21	14	23	13	17	68	1	116
S08	2009GZ11	9	13	18	19	13	3	18	3	21	14	23	13	17	68	1	116
S09	2009GZ12	9	13	18	19	13	3	18	3	21	14	23	13	17	68	1	116
S10	2009GZ13	9	13	10	19	13	3	18	3	21	14	23	13	17	68	1	140
S11	2009GZ14	9	13	18	19	13	3	18	3	21	14	23	13	17	68	1	116
S12	2009GZ16	9	13	18	19	13	3	18	3	21	14	23	13	17	68	1	116
S13	2009GZ17	9	13	18	19	13	3	18	3	21	14	23	13	17	68	1	116
S14	2009GZ18	9	13	18	19	13	3	18	3	21	14	23	13	17	68	1	116
S15	2009GZ19	9	13	4	19	13	3	18	3	21	14	23	13	17	68	1	123
S16	2009GZ20	9	13	4	19	13	3	18	3	21	14	23	13	17	68	1	123
S17	2009GZ21	9	13	18	19	13	3	18	3	21	14	23	13	17	68	1	116
S18	2009GZ22	9	13	18	19	13	3	18	3	21	14	23	13	17	68	1	116
S19	2009GZ24	9	13	18	19	13	3	18	3	21	14	23	13	17	68	1	116
S20	2009GZ26	9	13	18	19	13	3	18	3	21	14	23	13	17	68	1	116
S21	2009GZ27	9	13	18	19	13	3	18	3	21	14	23	13	17	68	1	116
S22	2009GZ30	9	13	18	19	13	3	18	3	21	14	23	13	17	68	1	116
S23	2009GZ31	9	13	18	19	13	3	18	3	21	14	23	13	17	68	1	116
S24	2009GZ32	9	13	18	19	13	3	18	3	21	14	23	13	17	68	1	116
S25	2009GZ33	9	13	18	19	13	3	18	3	21	14	23	13	17	68	1	116
S26	2009GZ34	9	13	18	19	13	3	18	3	21	14	23	13	17	68	1	116
S27	2009GZ35	9	13	18	19	13	3	18	3	21	14	23	13	17	68	1	116
S28	2009GZ37	9	13	18	19	13	3	18	3	21	14	23	13	17	68	1	116
S29	2009GZ38	9	13	171	19	13	3	18	3	21	14	23	13	17	68	1	141
S30	2009GZ39	9	13	18	19	13	3	18	3	21	14	23	13	17	68	1	116
S31	2009GZ41	9	13	18	19	13	3	18	3	21	14	23	13	17	68	1	116
S32	2009GZ42	9	13	18	19	13	3	18	3	21	14	23	13	17	68	1	116
S33	2009GZ43	9	13	18	19	13	3	18	3	21	14	23	13	17	68	1	116
S34	2009GZ44	9	13	171	19	13	3	18	3	21	14	23	13	17	68	1	141
S35	2009GZ45	9	13	18	19	13	3	18	3	21	14	23	13	17	68	1	116
S36	2009GZ46	9	13	171	19	13	3	18	3	21	14	23	13	17	68	1	141
S37	2009GZ47	9	13	18	19	13	3	18	3	21	14	23	13	17	68	1	116
S38	2009GZ48	9	13	4	19	13	3	18	3	21	14	23	13	17	17	1	76
S39	2009GZ49	9	13	4	19	13	3	18	3	21	14	23	13	17	17	1	76
S40	2009GZ50	9	13	4	19	13	3	18	3	21	14	23	13	17	17	1	76
S41	2009GZ51	9	13	4	19	13	3	18	3	21	14	23	13	17	17	1	76
S42	2009GZ53	9	13	4	19	13	3	18	3	21	14	23	13	17	17	1	76
S43	2009GZ54	9	13	4	19	13	3	18	3	21	14	23	13	17	17	1	76
S44	2009GZ55	9	13	4	19	13	3	18	3	21	14	23	13	17	17	1	76
S45	2009GZ56	9	13	4	19	13	3	18	3	21	14	23	13	17	17	1	76
S46	2009GZ57	9	13	4	19	13	3	18	3	21	14	23	13	17	17	1	76
S47	2009GZ58	9	13	4	19	13	3	18	3	21	14	23	13	17	17	1	76
S48	2009GZ59	9	13	4	19	13	3	18	3	21	14	23	13	17	17	1	76
S49	2009GZ61	9	13	4	19	13	3	18	3	21	14	23	13	17	17	1	76
S50	2009GZ62	9	13	4	19	13	3	18	3	21	14	23	13	17	17	1	76
S51	2009GZ63	9	13	4	19	13	3	18	3	21	14	23	13	17	17	1	76
S52	2009GZ64	9	13	4	19	13	3	18	3	21	14	23	13	17	17	1	76
S53	2009GZ65	9	13	4	19	13	3	18	3	21	14	23	13	17	17	1	76
S54	2009GZ66	9	13	4	19	13	3	18	3	21	14	23	13	17	17	1	76
S55	2009GZ69	9	13	18	19	13	3	18	3	21	14	23	13	17	17	1	122
S56	2010GZ07	9	13	4	19	13	3	18	3	21	14	23	13	17	17	1	76
S57	2010GZ08	9	13	4	19	13	3	18	3	21	14	23	13	17	17	1	76
S58	2010GZ09	9	13	4	19	13	3	18	3	21	14	23	13	17	17	1	76
S59	2010GZ10	9	13	4	19	13	3	18	3	21	14	23	13	17	17	1	76
S60	2010GZ11	9	13	4	19	13	3	18	3	21	14	23	13	17	17	1	76
S61	2010GZ12	9	13	4	19	13	3	18	3	21	14	23	13	17	17	1	76
S62	2010GZ13	9	13	4	19	13	3	18	3	21	14	23	13	17	17	1	76
S63	2010GZ14	9	13	4	19	13	3	18	3	21	14	23	13	17	17	1	76
S64	2010GZ15	9	13	4	19	13	3	18	3	21	14	23	13	17	17	1	76
S65	2010GZ16	9	13	4	19	13	3	18	3	21	14	23	13	17	17	1	76
S66	2010GZ17	9	13	4	19	13	3	18	3	21	14	23	13	17	17	1	76
S67	2010GZ18	9	13	4	19	13	3	18	3	21	14	23	13	17	17	1	76
S68	2010GZ19	9	13	4	19	13	3	18	3	21	14	23	13	17	17	1	76
S69	2010GZ20	9	13	4	19	13	3	18	3	21	14	23	13	17	17	1	76
S70	2010GZ21	9	13	4	19	13	3	18	3	21	14	23	13	17	17	1	76
S71	2010GZ22	9	13	4	19	13	3	18	3	21	14	23	13	17	17	1	76
S72	2010GZ23	9	13	4	19	13	3	18	3	21	14	23	13	17	17	1	76
S73	2010GZ24	9	13	4	19	13	3	18	3	21	14	23	13	17	17	1	76
S74	2010GZ25	9	13	4	19	13	3	18	3	21	14	23	13	17	17	1	76
S75	2010GZ26	9	13	4	19	13	3	18	3	21	14	23	13	17	17	1	76
S76	2010GZ27	9	13	4	19	13	3	18	3	21	14	23	13	17	17	1	76
S77	2010GZ28	9	13	4	19	13	3	18	3	21	14	23	13	17	17	1	76

Note: 15 loci including arcA, aroE, aspC, clpX, cyaA, dNaG, fadD, grpE, icdA, lysP, mdh, mtlD, mutS, rpoS and uidA and the primers can be accessed on the sharing EcMLST website (http://www.shigatox.net/ecmlst/cgi-bin/index). Each allele was assigned a different allele number and the allelic profile (string of fifteen integers) was used to define the sequence type (ST) based on the EcMLST website, which was established to provide a resource to investigators who can use this to assign the ST of further strains. New allele numbers and sequence type were submitted to the EcMLST curator for allocation. ST = Sequence Type.

## Discussion

*S*. *flexneri* was the predominant species in Guizhou Province in 1970s and 1980s, followed with *S*. *sonnei* [10], but *S*. *sonnei* is becoming the most important causative agent for shigellosis in recent decades in Guizhou Province [11]. No data, however, are available about the molecular characterization of the local isolates of *S*. *sonnei*. In order to systematically understand the molecular characteristics, four local isolates of *S*. *sonnei* from patients of shigellosis in 1974–1982 and 72 isolates from patients of shigellosis in 2008–2010 were used for analysis in this study. The serotypes of the isolates used in this study including two isolates of phase I and 74 isolates of phase II. The locality origin of these isolates covers five prefectures of the nine Profectures in Guizhou Province.

PFGE is a broadly applicable typing method with a high degree of intra- and inter laboratory reproducibility when standardized protocols are followed [13]. It was proved to be a powerful tool in the laboratory for discriminating *Shigella* isolates during an outbreak [28]. In this study, PFGE discriminate the 76 isolates of *S*. *sonnei* into 38 PTs, which indicates it is a powerful tool in discriminate *S*. *sonnei i*solates. PFGE based clustering tree branched out extensively, which suggests that the 76 isolates of *S*. *sonnei* in this study were heterogeneous.

The dentrogram of all the 76 isolates showed a 65% coefficient of similarity, which shared similar discriminatory power with *S*. *sonnei* isolates from other provinces [29, 30] and other countries [3, 31]. Isolates from close years were almost clustered closely and further divided into sub-clusters based on the region of isolation (Fig 1), indicating the genetic diversity and the genetic changes over the timescales.

MLVA is a prominent typing tool which has been developed for *S*. *sonnei* [2, 3, 15, 30]. In the present study, the 76 *S*. *sonnei* isolates were discriminated into 19 different MTs and showed a low (about 25%) coefficient of similarity, indicating the high discriminatory power of MLVA in subtyping *S*. *sonnei* isolates. Compared with the seven loci-based MLVA typing data of isolates from other conutries such as Malaysia[3], Japan [2], isolates of this study shared a lower overall similarity, which indicated the greater diversity and unique characteristics of *S*. *sonnei* isolates of Guizhou Province. Phylogenetic tree based on MLVA indicated that majority of isolates were clustered in accordance with the origin of isolation years, which were further closely clustered based on the geographical origin (Fig 2). The MST based on the MLVA data revealed two MCs and 11singletons among the 76 isolates, which further displayed the clonal relationships in the local isolates of Guizhou Province (Fig 3).

MLST based on 15 house-keeping genes were applied in *Shigella* genotyping and the protocol is shared at the EcMLST website [23, 24]. In this study, by using 15 house-keeping gens, 76 local isolates of *S*. *sonnei* from Guizhou Province, were divided into six STs, of which 4 STs including ST122, ST123, ST140 and ST141 were novel. Clustering tree based on the MLST data shows that all the 76 isolates were divided into two gross clusters, with a high coefficient of similarity (>90%), indicating the low discriminative ability in subtyping *S*. *nnei* isolates. Besides, MST based on the eBURST algorithm revealed that all the 76 isolates belong to only one CC with no singletons, indicating the relatively close genetic relationship among these isolates.

Additionally, according to the genotyping results of local isolates in this study, both of PFGE and MLVA displayed excellent discriminative ability in subtyping the *S*. *sonnei* isolates from Guizhou Province. Both techniques further differentiated the *S*. *sonnei* Phase I and II strains. However, MLVA was better at grouping the strains on the basis of isolation years, and the overall percentage of similarity among strains was low when subtyping using MLVA, which suggested MLVAwas of more excellent discriminative ability than PFGE in subtyping the *S*. *sonnei* isolates. Furthermore, MLVA subtyping was more rapid and less laborious compared to PFGE. Interpretation of result was less subjective and results were more readily comparable between laboratories. Compared with PFGE and MLVA, MLST divided the 76 isolates in to six STs, and all the STs belonged to one CC, which indicates the poor discriminative ability in distinguishing *S*. *sonnei*. All these suggest that MLVA may be a suitable complement to PFGE or even an alternative for routine subtyping of *S*. *sonnei*, and MLVA which is consistent with the early study by Koh, *et al*. in Malaysia [3], and use of a combination of molecular typing techniques is effective for epidemiological investigation.

## Conclusions

In the present study, we apply PFGE, MLVA and MLST methods to genetically characterize the 76 local isolates of *S*. *sonne* isolated in Guizhou Province in different years. PFGE based on XbaI digestion divided the 76 isolates into 38 PTs and MLVA based on seven VNTR loci discriminated them into 19 different MTs, while MLST based on 15 loci differentiated the isolates into six STs, with four STs being novel. Phylogenetic tree based on the three genotyping methods showed that majority of isolates were clustered in accordance with the years of isolation, which were further closely clustered based on the geographical origin. PFGE and MLVA displayed more excellent resolution than MLST in discriminating the isolates and reveal the changing of genetic characteristics over different rears. Our study enhances our understanding of genetic characteristics of *S*. *sonnei* in Guizhou Province, and provides a scientific basis for control and prevention of Shigellosis in the locality.
